# Exploring barriers to the use of formal maternal health services and priority areas for action in Sidama zone, southern Ethiopia

**DOI:** 10.1186/s12884-018-1721-5

**Published:** 2018-04-12

**Authors:** Aschenaki Z. Kea, Olivia Tulloch, Daniel G. Datiko, Sally Theobald, Maryse C. Kok

**Affiliations:** 1 REACH Ethiopia, P.O. Box 303, Hawassa, Ethiopia; 2 Liverpool School of Tropical Medicine, Liverpool, UK; 3 Royal Tropical Institute, P.O. Box 95001, 1090 HA Amsterdam, The Netherlands

**Keywords:** Maternal health, Three delays, Service utilization, Health extension workers, Ethiopia, Quality improvement

## Abstract

**Background:**

In 2015 the maternal mortality ratio for Ethiopia was 353 per 100,000 live births. Large numbers of women do not use maternal health services. This study aimed to identify factors influencing the use of maternal health services at the primary health care unit (PHCU) level in rural communities in Sidama zone, south Ethiopia in order to design quality improvement interventions.

**Methods:**

We conducted a qualitative study in six *woredas* in 2013: 14 focus group discussions (FGDs) and 44 in-depth interviews with purposefully selected community members (women, male, traditional birth attendants, local *kebele* administrators), health professionals and health extension workers (HEWs) at PHCUs. We digitally recorded, transcribed and thematically analysed the interviews and FGDs using Nvivo. The ‘three delay model’ informed the analytical process and discussion of barriers to the use of maternal health services.

**Results:**

Lack of knowledge on danger signs and benefits of maternal health services; cultural and traditional beliefs; trust in TBAs; lack of decision making power of women, previous negative experiences with health facilities; fear of going to an unfamiliar setting; lack of privacy and perceived costs of maternal health services were the main factors causing the first delay in deciding to seek care. Transport problems in inaccessible areas were the main contributing factor for the second delay on reaching care facilities. Lack of logistic supplies and equipment, insufficient knowledge and skills and unprofessional behaviour of health workers were key factors for the third delay in accessing quality care.

**Conclusions:**

Use of maternal health services at the PHCU level in Sidama zone is influenced by complex factors within the community and health system. PHCUs should continue to implement awareness creation activities to improve knowledge of the community on complications of pregnancy and benefits of maternal health services. The health system has to be responsive to community’s cultural norms and practices. The mangers of the woreda health office and health centres should take into account the available budgets; work on ensuring the necessary logistics and supplies to be in place at PHCU.

**Electronic supplementary material:**

The online version of this article (10.1186/s12884-018-1721-5) contains supplementary material, which is available to authorized users.

## Background

Globally, about 3 million maternal deaths occurred in 2015. The estimated maternal mortality ratio (MMR) for high income regions was 12/100,000 live births and, for low income regions was 239/100,000. The estimated MMR for Ethiopia is 353 /100,000 live births. The sustainable development goals (SDGs) call for an accelerated reduction in maternal deaths, so that the global MMR falls to 70 or below by 2030, working towards a vision of ending all preventable maternal mortality [1].

Use of maternal health services is instrumental for preventing maternal mortality and morbidity. Factors influencing the use of maternal health services have been the subject of research worldwide. In 1994, Thaddeus and Maine devised a model to categorize the main factors shaping three phases of delay in accessing services. Phase I presents the delay in the decision to seek medical care, including a range of factors at the individual level, driven by culture, illness characteristics, costs and previous experiences with the health care system. Phase II includes physical accessibility factors, such as the distribution of facilities, travel time to the facility, availability, and costs of transport and the condition of roads. Phase III is the provision of quality care: factors here include adequacy of the referral system; supplies and equipment and the availability and competence of health workers [2].

Studies, such as those carried out in Uganda, Pakistan, Myanmar, Egypt, and Ethiopia, have applied the model to examine and describe the three delays leading to maternal mortality [3–8]. These studies identified that factors which interplay at the first delay level contributed to maternal mortality, followed by the third delay factors which are associated with lack of adequate and quality care. Other research conducted in Brazil, Tanzania and Ethiopia used the model to assess the three delays in the provision of emergency obstetric care [9–11]. Studies done in Nicaragua and Ethiopia used the model to assess factors that affect women’s health seeking behaviour during pregnancy and delivery [12–15]. Also in Tanzania a study has been done using the three delays in relation to perinatal mortality [16]. In Ethiopia, two studies used the three delays framework to investigate the reasons for high perinatal deaths among newborns delivered in health facilities [8, 17]. These studies demonstrate that socio-demographic and cultural factors, perceived quality of care and previous maternal health care experiences affect health seeking behaviour of pregnant women.

In recent years, Ethiopia has invested in the expansion of health facilities as well as the training and deploying of community and mid-level health workers such as health extension workers (HEWs), public health officers, midwives and nurses, to improve access to health care. Primary health care is delivered through the *woreda* (district) health system comprising a primary hospital, 6–10 health centres and satellite health posts which together form a primary health care unit (PHCU). Maternal health services provided by a PHCU; range from simple preventive services at health posts to comprehensive maternal health services and emergency obstetric care at primary hospitals. Health centres provide comprehensive maternal health services and basic emergency obstetric services [18]. Health posts are staffed with two HEWs, who are female community health workers recruited from the community (*kebele*)^,^^1^ delivering 16 primary health care packages at the community level. HEWs are trained for 1 year and provide maternal health services like antenatal care (ANC), “safe and clean”^2^ delivery services and postnatal care (PNC) [19]. Health centres provide technical and administrative supervision to HEWs and the *woreda* health office has the general oversight of the health centres and health posts.

The government of Ethiopia promotes skilled birth attendance; HEWs should refer pregnant women to the health centre or primary hospital, especially in cases where there is a high risk or complications [19]. HEWs generally have good connections in their communities and work together with volunteers who are part of the health development army (HDA). The HDA is a formal system of community-based model families trained by the HEWs to deliver health extension packages independently [20], they are also referred to as the “1 to 5 network”.

Despite the expansion of primary health care delivery through PHCUs, the use of maternal health services remains low, which contributes to the country’s high MMR. In 2014, 41% of mothers made one ANC visit during pregnancy, 16% gave birth by a skilled provider and 13% received PNC within the first 2 days of delivery [21]. Official data suggest that, of the total women receiving ANC, 41% received ANC from nurses or doctors and 18% from a HEW. Over three quarters of all births were assisted by a relative, a traditional birth attendant (TBA) or another non-skilled person. About 2% of births were assisted by a HEW [21].

In Ethiopia, studies have identified factors that influence the use of maternal health services. Pregnant women who lived in urban areas, were literate, had approval from their husbands and families and had good knowledge of the danger signs of pregnancy were more likely to use maternal health services [22–26]. These studies were primarily quantitative and did not capture in-depth perspectives of mothers and other key stakeholders or focus on the PHCU. Given the rich cultural and linguistic diversity within Ethiopia [27, 28], there is need to understand the factors influencing use of maternal health services at community level within very specific cultural contexts, in order to develop locally appropriate interventions. This in-depth qualitative study explored, from multiple perspectives, factors affecting maternal health service use in rural Sidama, to provide context-specific information on experiences and delays in seeking care and to support the design of interventions focused on improving maternal health outcomes.

## Methods

### Study setting

The study was conducted in Sidama zone of the South Nation Nationalities and Peoples Region of Ethiopia in 2013. The capital of the zone is Hawassa. The zone comprises 19 rural *woredas* with a total population of 3.4 million. In Sidama, 12% of pregnant women gave birth assisted by a skilled birth attendant, 88% made one ANC visit, 48% made four ANC visits and 58% received PNC in 2013. Around 14% of births were assisted by HEWs [29].

### Study design, population, and sampling

This explorative qualitative study involved focus group discussion (FGDs) and in-depth interviews (IDIs). FGDs were held to understand the views and norms of different groups regarding factors influencing use of maternal health services. Interviews were carried out with community members and health professionals who were expected to be knowledgeable, have experience or were directly involved in services delivery at the primary health care unit.

A total of 14 FGDs were conducted: 6 FGDs with HEWs (one in each *woreda*), 6 FGDs with women (one in each *woreda*) and two FGDs with male (in two purposively selected *woreda* amongst the six).

We conducted a total of 44 IDIs: 12 IDIs with pregnant women or women who delivered recently (two in each *woreda*), 12 IDIs with HEWs (two in each *woreda*), 6 IDIs with TBAs (one in each *woreda*), 3 IDIs with *Kebel*e administrators (in three purposively selected *woredas*), 6 IDIs with persons in charge of the health centre or leaders of the labour room workers (one in each *woreda*), 3 IDIs with *woreda* health extension program coordinators (in three purposively selected *woredas*), 1 IDI with the zonal health department health extension program (HEP) coordinator and 1 IDI with the regional health bureau HEP coordinator (Table 1).

**Table 1:** An overview of participants in IDIs and FGDs

Level	Type of participants	Method	Number	Total
Community	Women	FGD	1 per woreda	6
	Male	FGD	In 2 woredas	2
	Women	Interview	2 per woreda	12
	TBA	Interview	1 per woreda	6
	Kebele administrator/Chair person	Interview	In 3 woredas	3
Health Workers	HEW	Interview	2 per woreda	12
	HEW	FGD	1 per woreda	6
	Person in charge of health centre / leader of labor room health workers	Interview	1 per woreda	6
HEP Coordinators	HEP Coordinator at woreda level	Interview	In 3 woredas	3
	HEP Coordinator at zonal health department	Interview	1 from zonal health department	1
	HEP Coordinator at regional health bureau	Interview	1 from regional health bureau	1
Total				58

We focused on the first two levels (health post and health centres) of the PHCU, as they are the first point of contact for the rural community to access maternal health services. Participants were purposefully selected eligible women comprised of those who had given birth in the previous 2 years or were pregnant at the time of data collection. Male participants for the FGDs were selected on the basis of having an influential position in the community: husbands (of women with babies), influential elders, religious leaders and other influential male in the community were included. HEWs and health professionals were those who served for three or more years so they had sufficient experience to share.

The study took place in six purposively selected *woredas*, taking into account the rate of skilled delivery in 2012 and the distance from the zonal capital. We took 12% skilled delivery, which was the average coverage of the zone in the year, as a cut off point to categorize the *woredas’* performance. The *woredas* achieving 12% or more were classified as “relatively well performing” and those below 12% were considered “poor performing”. We included three *woredas* which were performing relatively well and three *woredas* which were performing poorly, and ensured that half were close to the regional capital Hawassa, and half further away to explore differences in level settings.

To identify and recruit participants for the study at *woreda* level, the research team approached the *woreda* health office, discussed the objectives of the study, the sampling approach and strategies to be used for identification of participants. *Woreda* health office, HEWs and *kebele* administration supported the identification and recruitment of women for the study. Health centre and *woreda* health office staff assisted in the identification of HEWs and health professionals for inclusion in the study, and HEWs were involved in the recruitment of TBAs and community representatives.

### Data collection

Data were collected by four Ethiopian researchers who had experience in conducting qualitative studies and were trained for 1 week. Semi-structured interview guides were developed in English, translated to *Sidamigna* and *Amharic* (Additional file 1), and back translated and checked for consistency by an independent person from Ethiopia, who was able to speak both languages.

The interview guides contained questions eliciting factors that facilitate or hinder the use of maternal health services at different levels. During the interview and FGDs, participants were asked about their knowledge on maternal health services, health seeking behaviour, the factors influencing decision-making process, experience with pregnancy, use of services, possible barriers to use of the services and perceptions of service quality. Background information of participants such as age, education level, roles and responsibilities, services years (for health workers) were gathered and queries were run to see their effect on responses of respondents.

The interview guides were piloted in one *woreda,* were not included in the analysis and the questions were modified where needed. Debriefing sessions were held daily to discuss key findings, identify saturation of themes, refine questions and support the quality of the research process.

The interviews were carried out in the usual work settings of participants: health posts, health centres, offices and homes (TBAs only). The FGDs were conducted at schools and *kebele* administration offices. The duration of the IDIs was 60–90 min and for the FGDs 90–130 min. The FGDs were conducted by two facilitators: one recorder and one discussion facilitator, whereas the IDIs were conducted by one interviewer.

### Data analysis

All interviews and FGDs were digitally recorded, transcribed and translated into English. A sample of transcripts was randomly checked against the recordings to support quality assurance. Key themes were identified and a coding frame was developed after reading the transcripts in two pairs of two researchers. The coding frame and analytical process was informed by the three delays model to enable demarcation of experiences and challenges at different levels [2]. Transcripts were coded using Nvivo (v.10) software. The coded transcripts were further analyzed and summarized in narratives for each theme and sub-theme. The three delays model was used to frame our findings, as the model supports critical and integrated analyses exploring how individual, community and health system related factors hinder the use of maternal health services. Study findings were presented, discussed and validated in stakeholders meetings at the *woreda* and zonal level.

## Results

**Fig. 1:**
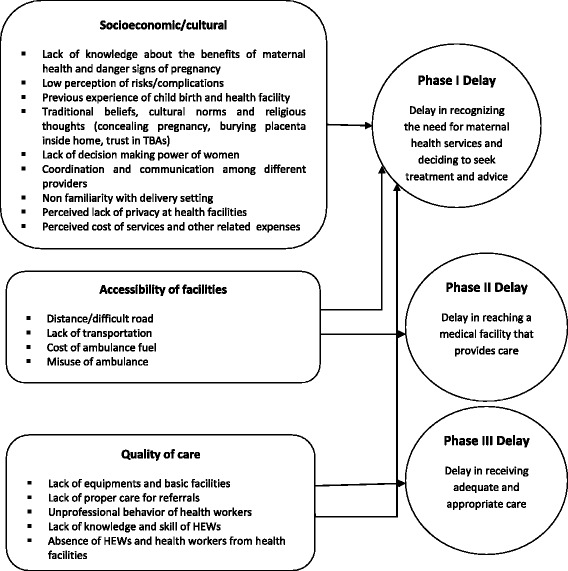
Applying the three delay model to understand factors limiting utilization of maternal health services in Sidama zone, Southern Ethiopia

The results are presented according to the different levels of the three delays model which informed our analytical process (Fig. 1) and supported by direct quotations from study participants.

Delay I: Delay in recognizing the need for maternal health services and deciding to seek treatment or advice.

### Lack of knowledge, perception of risks and previous childbirth and health facility experience

HEWs and male FGD participants described how some women chose not to use maternal health services because they are neither aware of danger signs during pregnancy and childbirth nor the benefits of obtaining maternal health services.

*“...lack of knowledge about the use and benefit of delivering at the health centre is the main reason; if she had the knowledge and awareness about the importance [of skilled delivery] she wouldn't have given birth at home.”* (HEW, interview).

Most of the HEWs and some *kebele* administrators supported the opinion that women had low perceptions of risks in pregnancy and therefore do not seek any maternal health services if they feel well during their pregnancy. Some women who participated in the study confirmed that they did not seek care unless they were sick and recognized there were complications; this was also confirmed by some male participants.

*“If there is no clear sickness, they [women] don’t come to visit health institutions.” (Male, FGD).*

Some women who had complications during previous pregnancies mentioned they had chosen to use routine maternal health services, while women who had experience with safe home delivery in the past seemed more likely to choose giving birth at home for subsequent deliveries.

*"They [women] want to give birth at their home. Especially those who experienced their previous labor at home say, ‘we were safe while we gave birth at home, so we don't want to come here (health post)’" (HEW, FGD).*

Some pregnant women reported that they did not plan to visit health facilities for their current pregnancy and would not recommend others to use maternal health services, as they felt they had not received proper care during their previous visit.

*“One time I (HEW) sent a pregnant woman who had some problems to the health centre. They sent her back without giving her any help. Another time I met this woman while I was telling another woman to go to the health centre to give birth. She said ‘I will never go there and don't recommend others to go.’” (HEW, FGD).*

### Traditional beliefs, religious practices, and cultural norms

There is a range of local behaviours that influence uptake of maternal health services. The tradition of concealing pregnancy during the early stages prevented some women from timely booking for ANC.

*"In our culture, we don't want to talk to people [about pregnancy]. People should know after the birth of the baby or when our abdomen becomes big. It is shameful and secret because we are not sure about the continuation of the pregnancy. There may be a miscarriage."* (Woman, interview).

In the community, giving birth at home was customary and women who gave birth at health facilities were considered weak. This may be related to the perception that health facilities are a place for managing severe illnesses rather than normal conditions like pregnancy and the fear that those women who visited health facilities were sometimes assumed to end up with surgical procedures or death.

*The first and the big challenge is traditional belief, they (women) say "my mum gave birth to eight of us at home, what happened to her? Even our grandma was delivering at home…"* (Interview, *woreda* HEP coordinator)

The culture of burying the placenta inside the home was reported as an important practice after the birth, and the worry that this may not be possible was an important deterrent factor against institutional delivery.

*"In the culture of this community they don't want to throw the placenta outside their home. Because of this, they don't want to go outside [to health facility] and lose the placenta. If the placenta is not buried inside the home it is considered as bad fortune for the baby. In the health facility, they don't get the placenta to take back to their home and consider it as a big loss, similar as if they lost the delivered baby."* (HEW, FGD).

Christian beliefs, for example, that God will help pregnant women during labour, were mentioned by the HEWs, male and female community members and influence health-seeking behaviour. It appeared to encourage women to deliver at home

*“The problem is, even though some people have awareness, they don’t want to show this in practice. They say ‘God will help a mother’”* (Interview, HEW)

The decision-making process of when and where a woman should get maternal health services was also influenced by family members, notably husbands and mother-in-laws. Women were expected to follow the same practice as their mothers and ancestors even if that was sometimes against their own preference.

“*...some women after being identified by voluntary community health workers [HDAs] don't want to come to the health post for ANC. They complain about their husband and mother-in-law: ‘My husband and mother-in-law don’t allow me to go for ANC.’”* (HEW, interview).

### Coordination of maternal health services with community-based providers

Most HEWs reported there was good coordination of maternal health services with HDA leaders, which increased use of services. HDAs are volunteers organized in a 1 to 5 group, with one leader. The role of HDAs is to support HEWs to perform health promotion activities and coordination of referral in the community. HEWs described how HDAs were involved in the identification of new pregnancies, counselling of mothers for ANC and health facility delivery and facilitation of referral of pregnant and laboring women.

*“We [HDAs] taught women in our community... the leaders of 1 to 5 networks give us the advice to convince pregnant mothers. When their labor starts we call to the health extension worker....”* (Woman, FGD).

Some HEWs mentioned that not all the HDA leaders were active in performing their tasks; this was partly due to the burden of housework, but also because they expected incentives from the government which they did not receive. In the past, some members of the HDAs had received incentives from government and non-governmental organizations during training and campaigns. Currently the government advocates not providing financial incentives for HDAs, given the large number of HDAs nationwide, for whom payment would be difficult to sustain.

Many participants reported that the role of TBAs has shifted. They are no longer formally supported by the government to provide delivery services; rather they are expected to support the referral of pregnant women to health facilities. Many community members supported the new roles of TBAs, however; the change in policy was said not to have been properly communicated to, nor acknowledged by the TBAs. Demand for TBA services persisted and some TBAs continued to provide delivery services in the community, as they were perceived to be more skilled and experienced than the HEWs and health professionals. There were different opinions from community members regarding HEWs’ skills: some described them as young and inexperienced, whilst others believed them to be better skilled than TBAs.

*“...TBAs receive only a little information [training] from the government but they are famous in the kebeles so the people say ‘the known devil is better than unknown God’ and the people believe in them [TBAs].”* (HEW, FGD).

*"...I never attended a mother after I got the order of the government. The health centre head warned us not to attend delivery. He said that you are not allowed to attend birth of any women, only to refer women to the health post and health centre."* (Interview, TBA).

*"...but after we were told to stop conducting delivery, I got disappointed, because we were not told what problems were created by us, they directly announced to stop our activities."* (Interview, TBA).

### Non-familiarity with delivery setting, health professionals, and privacy

For some women, visiting unfamiliar settings and not wanting to expose their bodies to unknown health professionals was a hindrance to their giving birth at health facilities. Women indicated that they would prefer their husband and relatives to be present at delivery rather than health workers. Another concern was that women would be expected to use the delivery bed, to lie down and stretch their legs, which differs from the customary squat position taken during birth at home. They believed it would expose them and make them vulnerable to illness. Some HEWs also mentioned women prefer female over male health professionals.

*"...the women hang their legs on the bed, which exposes them to “Qilleessa” bad wind which makes the women get sick and be in pain."* (HEW, interview)

### Perceived costs

Several types of cost had an impact on the decision of where to give birth. Anticipating costs or confusion about which services should be free at the primary hospital was a cause for concern. Indirect costs for food, transport and accommodation associated with a facility delivery also influenced the willingness of women and their families to accept referrals made by HEWs.

*"…. I paid in the hospital for the medical card and operation service. I paid around 350 birr in the hospital for all the services (drugs, bed and operation) for my first pregnancy."* (Woman, interview).

Some participants complained that payment was required by the hospital and this is against the rule of free maternal health service provision.

*“...the government stated [has a policy] maternal health services are to be provided free of charge, but women pay at hospital for delivery services, so I [HEW] stopped referring mothers to this hospital.” (HEW, FGD).*

Ambulances were available and are, in theory, free, but some participants had payment requested for ambulance fuel. This was a hindrance to some taking up referral and is closely linked to the second delay.

Delay II: Delay in reaching a health facility.

### Distance and transport problems

Accessibility of maternal health services at distant health facilities was problematic for some communities, this was characterised by the difficult terrain and large size of the geographical area served by a health facility. In the absence of transport, the community sometimes resorted to using locally made stretchers, “*kareza*”, to carry pregnant women.

*"...there is a place where it's hard to go even by motorbike. There are occasions where pregnant women have to walk 2 to 3 days to reach here. I met a mother who took three days... the biggest problem in this area is the problem of road and transport, while she was travelling here she gave birth on the road..."* (Delivery case team leader, interview).

Some areas reported poor availability of the *woreda* ambulance; this was due to an array of reasons, for example, appropriation of the ambulance by officials, drivers being uncooperative, or lack of fuel. Ambulance services were more likely to be impeded when called to areas where the topography and roads were difficult.

*"... We observe that mothers who live in a challenging geographical area don't come for institutional delivery. If mothers who live in a good geographical area call for an ambulance, they will benefit from the service..."* (HCH, interview).

Delay III: Delay in receiving adequate care.

### Availability of logistics, equipment, and basic facilities

Most participants felt that the local health posts and some health centres did not have the necessary medical equipment and basic facilities needed for provision of maternal health services. Lack of supplies appeared to affect motivation and performance of health professionals and HEWs as well as women’s willingness to seek services from these facilities.

*"We have a newly built health centre but nothing is supported there [no medical equipment], there are not even enough health professionals.... when there is a referral case from our health post but the health centre is not giving better services, then what is the difference between a health post and health centre? They have been using local "Kuraz" lighting [kerosene] as they do not even have a generator."* (HEW, FGD).

### Lack of proper training, skills and adequate care from health workers and HEWs

HEWs mentioned that some women who had been referred to facilities from the community had negative experiences, as the health professionals either did not respect them or provide quality health care. Part of the reason for this appeared to be the poor skills level and training of health workers at health centres.

*“...the major problem I faced is at the health centre. They are not handling our referrals properly. I had an experience: I sent a woman to the health centre. They sent her back to her home, but she delivered on the road without getting support.”* (HEW, FGD).

The quality of care at community level was criticised. *Woreda* HEP coordinators felt that the HEWs were not providing focused ANC, nor including all the components of care. This opinion was supported by others who felt that counselling by the HEWs during ANC visits was insufficient to encourage women to choose skilled attendance at delivery. HEWs themselves were critical about the quality of the delivery training they received to conduct safe and clean deliveries at health posts. Some reported having forgotten the basic delivery skills, because their training had no practical element, and they had little on-the-job experience partly due to a lack of necessary equipment for delivery at the health post. They also felt that the refresher training given to them had not improved their skills and confidence.

*“We took delivery training in our initial training, but we forgot all things about it since we were not doing it in the health post.” (HEW, FGD).*

### Availability of staff at health facilities

The absence of HEWs from health posts is a common problem creating an unreliable supply of health services. Health posts should be staffed by two HEWs, but this is often not the case. If a HEW is on leave or attending training, the health post can be closed for days or weeks. Some women noted that they had not received full ANC and other maternal health services due to irregular opening hours of the health posts. For example:

*"I didn’t receive ANC until I gave birth, because I repeatedly went there (health post) and sometimes she (HEW) was not available... mostly the health post doesn’t open for the beneficiary, it opens when they (the HEWs) have a meeting with the HDA and at the day of vaccinations."* (FGD-Women).

## Discussion

This is the first qualitative study to assess barriers to use of maternal primary health care services within the specific cultural context of Sidama. Below we synthesize the key findings under each level of delay, which aspects are unique to Sidama and then highlight the priorities for action to improve use of maternal health services and, ultimately, maternal health outcomes.

### First phase delay

There were multiple intersecting factors that constituted the first delay within Sidama: lack of knowledge of the benefits of maternal health services and the danger signs of pregnancy, cultural and traditional beliefs that are specific to Southern Ethiopia, complex decision-making processes shaped by gendered interactions between husband and families, worry about culturally inappropriate practices in health facilities, and perceived costs of services. HDA volunteers – coordinated by HEWs – played an important role in addressing some of these barriers.

Studies carried out in Sekela and Banja districts of Amhara regional state and Sodo town of the Southern region of Ethiopia have also shown that women who lacked awareness and understanding of the benefits of maternal health services and danger signs of pregnancy and childbirth were also less likely to use the services [23, 24, 30]. Studies conducted in Ethiopia, Nigeria, and Bangladesh have shown that the tradition of hiding pregnancy at early stage affects timely booking for ANC [31–33]. It is not uncommon for Ethiopian pregnant women to book during their second or third trimester [21, 23, 25, 34, 35]. The main reason for not attending early ANC or publically revealing early pregnancy is a feeling of embarrassment experienced by pregnant women, because it is presumptuous to talk of pregnancy when miscarriage cannot be ruled out. It is important to consider how these social norms can be addressed with sensitivity so that complications are detected early and managed appropriately [36]. The culture of burying the placenta inside the home for good fortune means that mothers are reluctant to give birth at the health facilities, as generally health facilities don’t provide the placenta for the families to take home. Similar findings have been observed in a study conducted in Kembata-Tembaro, South Ethiopia [37].

The place and time a woman should get maternal health services is influenced by a collective decision of the husband, mother-in-laws and other family members, and compounded by gender dynamics and poverty: not only are women often reliant on men for financial resources, but the local cultural norms mean that the views of mothers-in-law in Sidama need to be respected. Studies conducted in other parts of the country have documented similar findings [22, 38, 39]. Women who were able to decide themselves or jointly decide with their husbands were more likely to use maternal health services than those who were not able to decide themselves [26, 35, 40, 41].

Despite the availability of health professionals and HEWs nearby at the PHCU, TBAs were often the preferred choice to attend delivery over locally-based formal providers. Previous studies carried out in Ethiopia have confirmed that the majority of home deliveries were assisted by TBAs [34, 38, 42]. The community trust in TBAs – they provide services within the home and engage in traditional practices such as burying placentas inside home – meant they were often the first choice to provide maternal health care. This preference was compounded by concerns by some community members about the experience of HEWs, who were believed to be younger and less competent than TBAs. Even though HEWs had been trained to provide safe and clean delivery service, they often lacked basic knowledge and skills to carry it out, which deterred the community to use their services. Inadequate trust in the HEWs’ capacity has been reported elsewhere in Ethiopia and may reflect a systemic problem with the HEW training package. In future the lack of trust related to delivery services might be less profound, since the government announced that HEWs should no longer be expected to conduct deliveries. In northern Ethiopia it was also found that HEWs had a low performance with respect to delivery and had poor knowledge about antenatal care counseling, danger signs and symptoms and complications of pregnancy [43].

Communication and coordination of maternal health services between the TBAs and the PHCU were limited. Other studies carried out in Afar regional state of Ethiopia recognized the importance of integrating TBAs with the formal health system in the light of human resource shortage for maternal health [44]. This is a challenging area given the government’s resistance to the official inclusion of TBAs, and the sudden not well-communicated ban of TBA services, which can result in conflicts of interest between TBAs and HEWs.

This study and a number of other Ethiopian studies identified that women often pay for delivery services at hospitals and for routine pregnancy investigations and care at health centres. A study carried out in Addis Ababa identified that 91% of women paid for investigations (laboratory and ultrasound) or drugs during ANC visits in government health facilities [45]. The Ethiopian essential service package policy indicates that delivery service should be provided free of charge [46]. Several studies have documented that user fees for maternal health service have an impact on service use [37, 39, 47–50]. Other costs for transportation, food and lodging form also as challenges for using health services [22, 37, 39, 47]. Exemption of service fees improves use of the services. For instance in Kenya, a four-fold increase in hospital deliveries was observed among a study population who had both the cost of services and transport covered compared with a population where their costs were not covered [48].

### Second phase delay

Lack of transport, distance, difficult topography, the cost of ambulance fuel, use of an ambulance for unintended purpose and uncooperative behavior of ambulance drivers affected the coordination of referrals within the PHCU and were the main factors with respect to delay two. Studies conducted in Ethiopia, Tanzania and Ghana have documented the problem of transport as a hindering factor for use of maternal health services [21, 22, 37, 39, 49, 50], but in this study, there are some specific hindrances that can be tackled locally. For example, some communities were affected by the improper use of the ambulance by local officials and the poor behavior of ambulance drivers.

### Third phase delay

Lack of medical equipment, weak coordination, and management for referrals, health workers’ unprofessional behaviour and absence of HEWs and health workers at health facilities were factors contributing to the third delay. This is consistent with other studies, one conducted in Tigray, Ethiopia, identifying that the majority of the health posts did not have medical equipment, water, and electricity supply [43].Other studies confirm that poor availability of drugs and supplies influences the use of maternal health services [37, 51, 52]. It will be necessary for health facilities and management teams in Sidama to address the poor availability of supplies and medical equipment at the PCHU if they are to increase the willingness of women to use formal local delivery services.

### Towards improving use of maternal health services in Sidama

This study identified a number of challenges that are specific to local circumstances and some are amenable to locally developed solutions. Special attention to these challenges and local solutions is instrumental, as the current interplay of different factors makes many pregnant women decide still to deliver at home. The REACH Ethiopia NGO, in collaboration with Sidama zone health department through the REACHOUT consortium, has been implementing a quality improvement intervention to start the process of addressing key factors influencing the use of maternal health services in PHCU which will be reported elsewhere. The intervention facilitated local identification of priority problems that would lead to activities to bring about small, non-costly incremental changes to try to overcome them. The quality improvement cycle approach can facilitate which priority actions are needed and feasible to support coordination of and engagement with on maternal health at the community level. This approach attempts to works within existing systems and funding constraints.

This study found targeted awareness creation and community mobilization activities are needed to improve the knowledge of pregnant women and their families on danger signs of pregnancy and the benefits of maternal health services. Pregnant women also need to be identified at earlier stage of pregnancy by the HDAs and HEWs to enhance timely booking for ANC. Community based health programmes should include husbands and mothers in laws so that they will be aware of maternal health issues in order to support pregnant women in getting the services. Local leaders need to communicate clear directions how the TBAs can contribute to maternal health services and optimize their coordination with HEWs. The activities of 1 to 5 network should be strengthened to enhance access to maternal health services within the community.

Action is also required to improve the responsiveness, quality and cultural sensitivity of health facilities, and women’s ability to get to these services. Ambulances should be used for the intended purpose and the government has to allocate a budget for fuel replenishment. The health system should take into account the cultural concerns of the community, like burying placenta inside home. Health facilities also need to be equipped with the necessary logistics and infrastructure, taking into account what is possible within available budgets. The government also needs to actively monitor the implementation of free maternal health services as per the regulation of the country.

Advocacy with local decision-makers and policy-makers on the importance and benefits of maternal health interventions, such as quality improvement cycles, is important for their sustainability. Ideally they need to be embedded in the health system from an early stage to ensure long term benefits.

### Limitations

This study was part of a larger study looking at factors influencing the performance of maternal health services delivered by HEWs. Use of maternal health services at the PHCU level was an important emerging theme and from the women’s perspective most factors related to delay 1 with relatively limited information on delays 2 and 3. Due to the recent ban on TBAs not to conduct delivery services, they were often reluctant to give detailed information.

## Conclusions

Maternal health remains a priority in Ethiopia and strategies to improve maternal health outcomes that are contextually relevant are urgently required. In this study, we have used qualitative research to identify key barriers constraining use of maternal health care services in PHCUs in rural communities in Sidama. These have been framed within Thaddeus and Maine’s three delays model; delays at the first and third phases emerged as particularly problematic in this study. There is a need to institute community based awareness creation and mobilization activities to address factors contributing to the first delay. Lack of logistics and supplies at health facilities and lack of proper training and skills of health workers need due attention from health system mangers and policy makers to address the third phase delay. The health system should also work towards more caring and compassionate health professionals. Action at these multiple intersecting levels will provide women with the quality and responsive health services they deserve to make progress towards the SDGs. One of the approaches that policy makers and local leaders can consider for doing this is quality improvement cycles.

## Additional file

Additional file 1: Interview guides on exploring barriers to the use of formal maternal health services and priority areas for action in Sidama zone, southern Ethiopia. (DOC 179 kb)

## Abbreviations

ANC: Antenatal care
FGD: Focus group discussion
HDA: Health development army
HEP: Health extension programme
HEWs: Health extension workers
IDI: In-depth interview
MMR: Maternal mortality ratio
PNC: Postnatal care
TBAs: Traditional birth attendants

## Footnotes

1 *Kebele* is the smallest unit of local government and can best be regarded as neighborhoods localized and delimited groups of people.

2 ‘Clean and safe’ deliveries are conducted by the HEW at the health post level and skilled deliveries are conducted by nurses and other health professionals at health centres and hospitals.
