# Supplementary material for: Exploring barriers to the use of formal maternal health services and priority areas for action in Sidama zone, southern Ethiopia
Source: BMC Pregnancy Childbirth. 2018 Apr 12;18:96. doi: 10.1186/s12884-018-1721-5 (PMC5897996; doi:10.1186/s12884-018-1721-5)
Supplement: Supplementary file 1 — Interview guides on exploring barriers to the use of formal maternal health services and priority areas for action in Sidama zone, southern Ethiopia. (DOC 179 kb) [file 12884_2018_1721_MOESM1_ESM.doc]

**REACH Ethiopia, REACHOUT Project Context Analysis Study In-depth Interview Topic Guide with Woman (IDI-WO)**

***Instruction for Interviewer***

1. Obtain consent
2. Fill in information sheet for each mother

**Information Sheet**

| Respondent identifier |  |
| --- | --- |
| Age |  |
| Name of village |  |
| Attended ANC? How many times? |  |
| How long ago last contact with HEW? |  |
| How often HDA contact with HEW in last 12 months? |  |
| Number of living children and age? |  |
| Position in HDA? (Leader or member) |  |
| How many pregnancies? |  |
| *If pregnant,* when is your baby expected? |  |

**Background of the interviewee**

1. Please tell us about yourself and your family.

What is your occupation? Who is the main income earner in your household? Do you have your own personal income? What is the household income? Who controls the household expenditure?

1. What is the level of your education?

**Critical incidence:**

Now we would like to speak with you about your own experience in pregnancy, we would like you to describe what happened in your most recent pregnancy from the time you knew you are pregnant, until the time you delivered your baby, (or in the case of pregnant women on first pregnancy) until now.

1. For the most recent pregnancy, how did you find out you were pregnant, and what did you do when you found out?

**Probes:** Did you recognize the signs; did you go for a test? If yes, who gave you the test? What did the person who gives the test say?

1. What did you do after you were sure you are pregnant?

**Probes:** told the husband, relatives, went to health post/ANC, changed normal behaviour

1. Did you do anything to make your pregnancy safe?

**Probes:** did you seek advice? From whom? (Relatives? TBA? HEWs? Health professionals? Other members of the community? Leader of HDA?) Why did you ask this person? What advice did they give you?

1. What did you do next? Did you follow their advice?
2. Did you go to the health centre during your pregnancy? Why/why not?

*If they went to the health centre,* Why did you go to the health centre? Did the HEW refer you or did you make the decision to go yourself? Why?

*If they have not yet mentioned ANC say:*

1. What do you think about ANC? Why did you go to ANC? / Or Why not? Do you think it is important?

**Probes:** Were you advised to go to ANC? By whom? What do you think about the ANC services? Why?

*If they received ANC:*

1. What happened at your ANC visits? How many did you have?
2. Last year there was a community mobilization about maternal health and institutional delivery, do you remember it? Can you remember talking with the HDA leaders about this?

**Probe:** Can you remember what they told you? What do you think about it?

1. For those who are HDA leaders: how did your community respond? How can we improve the messages to families about maternal health?

*Only for women who have more than one pregnancy before:*

1. How did your recent experiences with ANC compare to your previous pregnancies? Did you have ANC for all of your pregnancies? What influenced your decisions for this?
2. What did you do when your (most recent) labour started?

**Probe:** who did you contact (HEW??TBA)? Why?

1. What happened next? Where did you give birth? Why did you decide to give birth there?

**Probes:** if at health centre, how did you get there? Who helped you? How long did it take? Were you referred (by whom?)Were there any problems? Were there any costs?

1. How long was your labour, and how did you feel about it at this time? Did you have any problems associated with your labour (abnormal bleeding, other complications)? If yes, what happened, and what did you do? Who advised you? Who helped you?

*Only for women who are pregnant right now:*

1. Where are you planning to give birth? Are there any things you will do differently compared to the other time(s) you were pregnant?
2. What happened after you gave birth?

**Probe:** What advice did you receive? (nutrition, hygiene, breast feeding, long term contraceptive, post partum IUCD, anything else), are you advised about family planning and spacing of children?

1. Who gave you advice after childbirth? When? Did anyone visit you? When?
2. You told me you have xx children? How do your experiences of pregnancy and childbirth compare? Have you ever experienced complications in previous pregnancies, how many of your children are living now? (If some are dead – at what age? If neonatal: what happened? Were you offered referral? Did you accept to be referred? Why?)
3. How did you feel about the advice and services that you received during your pregnancy, during labour and after labour (PNC)?

**Probe**: Which services do you think are useful during pregnancy? What do you think can be done to improve the maternal health services?

***All women can be asked (first pregnancy and women with more than one pregnancy) (Q21-26)***

1. Who do you feel should be present when you give birth? (Probe: family, TBA, HEW, midwife?) Why?

**Probe:** comfort, profession person with skills in childbirth)

1. Whose advice do you trust the most about your pregnancy?

HEW? TBA? Midwife? Relative? Other health professionals or HDA? Why?

1. How do you feel about the attitude AND PERFORMANCE of the providers (HEWs/HPs like midwives)?
2. What could be improved? Which ones are the most useful for you:

**Probe:** e.g. have the best skills and knowledge? Convenient? Close to home? Useful. Which ones have limitations? Why?

1. Have you experienced any costs for maternal health services?

**Probes:** what for? Tests? Drugs? Consultation fees (from TBA or HEW?), transport? Other?

*If no,* do you know if other people sometimes are asked to pay? Do you think that service payment have an impact on the service utilization?

*If these areas have not yet been covered ask:*

1. What do you think is expected from the Government to improve the maternal health services (probe at levels: health facilities, district health office and zone health department)?

**REACH Ethiopia, REACHOUT Project Context Analysis Study In-depth Interview Topic Guide with Health Extension Worker (IDI-HEW)**

***Instruction for Interviewer***

1. Take individual consent
2. Fill in information sheet for each respondent

***For this interview maternal health services refer to ANC, delivery and post-partum services including FP.***

| Respondent identifier |  |
| --- | --- |
| What is your age? |  |
| How long have you been a HEW |  |
| Did you get additional training since you qualified? | For what: How long: |
| Where do you live? |  |
| Do you work in your home kebele? If not how far is your home? |  |

**Introduction**

- 1. Please introduce yourself, and how did you become HEW.

1. What made you decide to join the programme? What attracted you?
2. What was the selection process for becoming HEW?
3. Who are your clients? Please draw a map of the kebele and show where the clients come from? Are there some areas where you have less contact with the community? Is it more difficult to provide services in some areas of the kebele? Why?

**Incentives and motivation**

- 1. You work on 16 health packages. Which packages do you like to do the most? Why?
  2. What things make you and other HEWs feel good or not so good about your work? Why?

**Probing,** salary, transfer, leave, housing condition, logistic supplies, personal development, working for the community, job security...etc.)

- 1. What other incentives do you have for your work

**Probe:** social status, livelihood support, economic benefits, other rewards? Concerns?

- 1. How could your daily tasks be improved?

Probe for equipment and supplies, workload, working environment, communication, equipment and transportation, safety, career perspective, supervision, community, clients, colleagues, other health workers.

- 1. Which of the 16 packages takes most of your time? Why?
  2. Do you have enough time to do all of the 16 packages in your routine? – are there times when you cover more than one task when you do house to house visits?

1. What are the things that you like about providing maternal health services? And what do you not like about maternal health services? Why?

**Supervision**

- 1. Who supports and supervises you? (Probe: Kebele leaders, HCs or Woreda Health Office?)
  2. How often do you receive supervision?
  3. How is the quality of your work evaluated? By whom? How? How do you feel about this? Do you get feedback?
  4. Do you have enough support and supervision? What could be better?
  5. Do you communicate with colleagues (including supervisors Kebele HCs and Woreda Health Office, volunteers and TBAs) and if yes, how does it help you to do your job?
  6. How do you ensure that the community is satisfied with your service?

**Probe:** do you evaluate yourselves, suggestion box or book. What do people suggest?

**Maternal health work**

- 1. What services do you currently provide for pregnant women and delivering mothers?
  2. How do you approach a pregnant woman? What do you do first?

**Probe:** do you go from home to home; do you wait until they visit you themselves?

- 1. What training and skills have you been given for maternal health? When did you get this training?
  2. Are you confident that the training is enough to provide a high quality of MH service? Why? In which area do you need more training (probe out of ANC, Delivery and PNC)?
  3. Who do you work with in delivering maternal health service? (Probe HCs, HEP coordinators...)

Probe who supervises and guides you in this work? How do they guide you? What additional guidance would help you in your work?

- 1. What are the challenges in implementing MH services?

Probe ANC, delivery, post- partum

- 1. What do you think goes really well in your maternal health work? Why?
  2. What do you think does not always go well? Can you give an example? What things are influencing that this work does not go well?
  3. Thinking about your work and what can be done to improve it what would you suggest? How could this be done?

**Referral**

- 1. What do you do when a client has a problem you cannot solve? Who do you refer to? How does the referral process work?
  2. What is the referral mechanism for high risk pregnant women?

**Probes:** Are there any difficulties in making referrals? (Transport, costs, referral system, distance, attitudes of clients?)

- 1. What goes well and not so well in referral? Why?

**Community attitudes to MH and health seeking**

- 1. How do you know what the community or clients think about the services you provide? What do they like best? What do they complain about?
  2. Who do women normally contact when they are pregnant? At what stage of pregnancy do they normally seek health advice?
  3. At what stage of pregnancy do you think that women should come for ANC?

**Probe:** Are there any difficulties in encouraging women to seek ANC? Which difficulties? How can they be encouraged to come at the right time? What does the community think about the need for MH services?

- 1. Where do people in this community feel they should go for delivery?

**Probe:** home? Health post? Health centre? Why? Attended by whom? TBA, HEW?

- 1. In this area the proportion of women receiving ANC is quite high, but the proportion of women choosing institutional delivery is low, why do you think this is?
  2. Do you face challenges in encouraging women that they should deliver in a health facility?

**Probe:** Why? What/who influences the decisions women make about their maternal health seeking?

- 1. How do you communicate with community members on maternal health issues?

**Probe:** contact with committees, kebele leaders, HDA, TBAs).

- 1. How could the communication be improved? **Probe**: who should be involved? If TBAs are mentioned: how do they work together, what is the advantage or disadvantage?
  2. Which are the challenges at community level which make women not access ANC/Delivery/Post-partum services?
  3. Last year the HDA did community mobilization to promote institutional delivery, how did they do this in your Kebele?
  4. What was the impact of the community mobilization?
  5. Do you have ideas for additional activities that would help?

**Probe:** what about increased communication and monitoring of pregnant women between HEWs and other maternal health services, using mobile phone technologies? What impact could this have on your work? Would you favour this type of intervention?

- 1. What can be done to improve women’s maternal health seeking?

**Probe:** trust, education, community activities

**Monitoring and evaluation, quality of care**

- 1. What records do you or others keep of your work? How is this information collected?
  2. What happens with this information? Do you get feedback about the results of your work?
  3. Do you use mobile phones for your work? What do you use it for?

**Probe**: for different use: to collect and send information; to coordinate things; to seek advice from others, to contact clients. For each find out what with who how often.

- 1. Who bought the device? Who pays for the costs of use, air time, charging etc.?
  2. How do you feel about the use of these devices: advantages, disadvantages?

**Probe:** Do you use it for your MH work?

- 1. Do you think there is potential for using mobile phones in your MH work? How?

**Probe**: what about for making referrals of high risk mothers, sending monitoring data? What sort of challenges do you think there will be?

**REACH Ethiopia, REACHOUT Project Context Analysis Study Focus Group Discussion Topic Guide with HEWs (FGD_HEW)**

***Instruction for Interviewer***

Take consent

Fill in information sheet

Explain process

Ensure that ground rules are discussed

**Information Sheet**

| Date: District: Location of FGD: | | | | |
| --- | --- | --- | --- | --- |
| Respondent ID | Responsible for which Kebele | Home kebele | Age | How many years as HEW |
|  |  |  |  |  |
|  |  |  |  |  |
|  |  |  |  |  |
|  |  |  |  |  |
|  |  |  |  |  |
|  |  |  |  |  |
|  |  |  |  |  |
|  |  |  |  |  |

**Introduction**

1. Please introduce yourself.

2. What made you decide to join the programme? What attracted you?

**Incentives and motivation**

1. You work on 16 health packages. Which packages do you like to do the most? Why?
2. What things make you feel good or not so good about your work? Why?

**Probing**, salary, transfer, leave, housing condition, logistic supplies, personal development, working for the community, job security...etc.)

1. What other incentives do you have for your work? **Probe:** social status, livelihood support, economic benefits, other rewards? Concerns?
2. How could your daily tasks be improved? **Probe**: for equipment and supplies, workload, working environment, communication, equipment and transportation, safety, career perspective, supervision, community, clients, colleagues, other health workers.
3. Which of the 16 packages takes most of your time? Why?
4. Do you have enough time to do all of the 16 packages in your routine? – are there times when you cover more than one task when you do house to house visits?
5. What are the things that you like about providing maternal health services? And what do you not like about maternal health services? Why?

**Supervision**

1. Who supports and supervises you? (Probe: Kebele leaders, HCs or Woreda Health Office?)
2. How often do you receive supervision?
3. How is the quality of your work evaluated? By whom? How? How do you feel about this? Do you get feedback?
4. Do you have enough support and supervision? What could be better?
5. Do you communicate with colleagues (including supervisors Kebele HCs and Woreda Health Office, volunteers and TBAs) and if yes, how does it help you to do your job?
6. How do you ensure that the community is satisfied with your service? **Probe:** do you evaluate yourselves, suggestion box or book. What do people suggest?

**Maternal health work**

1. What services do you currently provide for pregnant women and delivering mothers?
2. How do you approach a pregnant woman? What do you do first? **Probe:** do you go from home to home; do you wait until they visit you themselves?
3. What training and skills have you been given for maternal health? When did you get this training?
4. Are you confident that the training is enough to provide a high quality of MH service? Why? In which area do you need more training (**prob**e out of ANC, Delivery and PNC)?
5. Who do you work with in delivering maternal health service? (**Probe** HCs, HEP coordinators...) **Probe:** who supervises and guides you in this work? How do they guide you? What additional guidance would help you in your work?
6. What are the challenges in implementing MH services? **Probe**: ANC, delivery, post- partum
7. What do you think goes really well in your maternal health work? Why?
8. What do you think does not always go well? Can you give an example? What things are influencing that this work does not go well?
9. Thinking about your work and what can be done to improve it what would you suggest? How could this be done?

**Referral**

1. What do you do when a client has a problem you cannot solve? Who do you refer to? How does the referral process work?
2. What is the referral mechanism for high risk pregnant women? **Probes:** Are there any difficulties in making referrals? (Transport, costs, referral system, distance, attitudes of clients?)
3. What goes well and not so well in referral? Why?

**Community attitudes to MH and health seeking**

1. How do you know what the community or clients think about the services you provide? What do they like best? What do they complain about?
2. Who do women normally contact when they are pregnant? At what stage of pregnancy do they normally seek health advice?
3. At what stage of pregnancy do you think that women should come for ANC? **Probe:** Are there any difficulties in encouraging women to seek ANC? Which difficulties? How can they be encouraged to come at the right time? What does the community think about the need for MH services?
4. Where do people in this community feel they should go for delivery? **Probe:** home? Health post? Health centre? Why? Attended by whom? TBA, HEW?
5. In this area the proportion of women receiving ANC is quite high, but the proportion of women choosing institutional delivery is low, why do you think this is?
6. Do you face challenges in encouraging women that they should deliver in a health facility?

**Probe:** Why? What/who influences the decisions women make about their maternal health seeking?

1. How do you communicate with community members on maternal health issues? **Probe:** contact with committees, kebele leaders, HDA, TBAs).
2. How could the communication be improved? **Probe**: who should be involved? If TBAs are mentioned: how do they work together, what is the advantage or disadvantage?
3. Which are the challenges at community level which make women not access ANC/Delivery/Post-partum services?
4. Last year the HDA did community mobilization to promote institutional delivery, how did they do this in your Kebele?
5. What was the impact of the community mobilization?
6. Do you have ideas for additional activities that would help? **Probe:** what about increased communication and monitoring of pregnant women between HEWs and other maternal health services, using mobile phone technologies? What impact could this have on your work? Would you favour this type of intervention?
7. What can be done to improve women’s maternal health seeking? **Probe**: trust, education, community activities

**Monitoring and evaluation, quality of care**

1. What records do you or others keep of your work? How is this information collected?
2. What happens with this information? Do you get feedback about the results of your work?
3. Do you use mobile phones for your work? What do you use it for? **Probe**: for different use: to collect and send information; to coordinate things; to seek advice from others, to contact clients. For each find out what with who how often.
4. Who bought the device? Who pays for the costs of use, air time, charging etc.?
5. How do you feel about the use of these devices: advantages, disadvantages? **Probe:** Do you use it for your MH work?
6. Do you think there is potential for using mobile phones in your MH work? How?

**Probe:** what about for making referrals of high risk mothers, sending monitoring data? What sort of challenges do you think there will be?

**REACH Ethiopia, REACHOUT Project Context Analysis Study Focus Group Discussion Topic Guide with Women (FGD_WO)**

***Instruction for Interviewer***

Take consent

Fill in information sheet

Explain process

Ensure that ground rules are discussed

**Information S**heet FGD-WO

| **Respondent ID** | **Age** | **Role in community or occupation** | **Education None/primary/**  **secondary/tertiary** |
| --- | --- | --- | --- |
|  |  |  |  |
|  |  |  |  |
|  |  |  |  |
|  |  |  |  |
|  |  |  |  |
|  |  |  |  |
|  |  |  |  |

**MH services**

1. What services are available for pregnant women in your kebele?

**Probe**: Where are these services: in health centres, health posts? Who provides these services (TBA? HEW?). When and how often do pregnant women receive these services?)

1. Are all the maternal health services that women need available in your community? What other services are needed?

**Perceptions about maternal health and health seeking behaviour**

1. In your community what does a woman normally do when she finds out she is pregnant?

**Probes:** Go to health post? Get a test? Where?

1. What do women do to make sure their pregnancy safe?

**Probes:** where can do they seek advice? From whom? (Relatives? TBA? HEWS? Health professionals? Other members of the community? Leader of HDA?) What sort of person is preferable?

1. Do you think is it important for pregnant women to attend antenatal care? Why? When do you think the first ANC visit should be? How many ANC visits should be done? Why?
2. What makes some women decide to attend ANC with health professionals, HEWs and others to seek advice from TBAs?

**Probe:** about family advice, costs, time, traditional beliefs etc.

1. What would encourage mothers to go for antenatal care with a HEW?

**Probes:** what are your friends and family members (mother, husband, mother in-laws and sisters) experiences and other women you know?

1. Why do you think some pregnant women in your community deliver in the health facilities? Why do other women decide to deliver at home? Do you think there is a difference in delivering at home and HC? What kind of difference?

**Probes:** if at health centre, how can you get there? Who helps you? How long does it take?

1. How does referral to the health centre happen (by whom?) are there any costs?
2. How can women be encouraged to give birth in a health facility?
3. Is advice available after childbirth?

From whom? When? What advice do they give?

**Probe:** What advice do women receive? (nutrition, hygiene, breast feeding, long term contraceptive, post partum IUCD, anything else), are you advised about family planning and spacing of children

1. Are there some women who don’t have access (physical, financial) to maternal health advice (from HEW or other providers)
2. Are there any costs for maternal health services?

**Probes:** what for? Tests? Drugs? Consultation fees (from TBA or HEW?), transport? Other? Do you think MH service cost have an impact on the utilization of service?

1. Whose advice do women trust the most about pregnancy?

HEW? TBA? Health professionals? Relative? Why?

1. Which ones are the most useful:

**Probe:** e.g. have the best skills and knowledge? Convenient? Close to home?

**Perceptions of HEWs service quality**

1. How do you feel about the maternal health services provided by HEW in this community? **Probe:** for availability, adequacy, distance, usefulness, and limitations. How do you feel about the skills and knowledge of HEWs? What are they good at? How are their maternal health skills?
2. How do you feel about their attitude towards the community? Ask for examples.
3. What would you like to see improved?
4. What services do you expect to be delivered to mothers by the HEWs?
5. Who do you feel should be present when you women give birth? (Probe: family, TBA, HEW, midwife?) Why?

**Probe:** comfort, profession person with skills in childbirth?

1. Last year there was a community mobilization about maternal health and institutional delivery, do you remember it? Can you remember talking with the HDA leaders about this?

**Probe:** Can you remember what they told you? What do you think about it?

For those of you who are HDA leaders: how did your community respond? How can we improve the messages to families about maternal health?

1. Do HEWs and other providers use mobile telephones or other technology to coordinate with health centres and other MH services? Do you think this important? Why?
2. What do you think the Government can do to improve the maternal health services (probe at levels: health facilities, district health office and zone health department)?
3. Do you think there are enough infrastructures, materials and logistic to give an adequate and high quality MH service at HP level?

**REACH Ethiopia, REACHOUT Project Context Analysis Study Focus Group Discussion Topic Guide with Male (FGD_Male)**

***Instruction for Interviewer***

Take consent

Fill in information and recording sheet

Explain process

Ensure that ground rules are discussed

**Information Sheet FGD-Male**

| **Respondent ID** | **Age** | **Role in community or occupation** | **Education None/primary/**  **secondary/tertiary** |
| --- | --- | --- | --- |
|  |  |  |  |
|  |  |  |  |
|  |  |  |  |
|  |  |  |  |
|  |  |  |  |
|  |  |  |  |
|  |  |  |  |

**MH services**

1. What services are available for pregnant women in your kebele?

**Probe**: Where are these services: in health centres, health posts? Who provides these services (TBA? HEW?). When and how often do pregnant women receive these services?)

1. Are all the maternal health services that women need available in your community? What other services are needed?

**Perceptions about maternal health and health seeking behaviour**

1. In your community what does a woman normally do when she finds out she is pregnant?

**Probes:** Go to health post? Get a test? Where?

1. What do women do to make sure their pregnancy safe?

**Probes:** where can do they seek advice? From whom? (Relatives? TBA? HEWS? Health professionals? Other members of the community? Leader of HDA?) What sort of person is preferable?

1. Do you think is it important for pregnant women to attend antenatal care? Why? When do you think the first ANC visit should be? How many ANC visits should be done? Why?
2. What makes some women decide to attend ANC with health professionals, HEWs and others to seek advice from TBAs?

**Probe:** about family advice, costs, time, traditional beliefs etc.

1. What would encourage mothers to go for antenatal care with a HEW?

**Probes:** what are your friends and family members (mother, husband, mother in-laws and sisters) experiences and other women you know?

1. Why do you think some pregnant women in your community deliver in the health facilities? Why do other women decide to deliver at home? Do you think there is a difference in delivering at home and HC? What kind of difference?

**Probes:** if at health centre, how can you get there? Who helps you? How long does it take?

1. How does referral to the health centre happen (by whom?) are there any costs?
2. How can women be encouraged to give birth in a health facility?
3. Is advice available after childbirth?
4. From whom? When? What advice do they give?

**Probe:** What advice do women receive? (nutrition, hygiene, breast feeding, long term contraceptive, post partum IUCD, anything else), are you advised about family planning and spacing of children

1. Are there some women who don’t have access (physical, financial) to maternal health advice (from HEW or other providers)
2. Are there any costs for maternal health services?

**Probes:** what for? Tests? Drugs? Consultation fees (from TBA or HEW?), transport? Other? Do you think MH service cost have an impact on the utilization of service?

1. Whose advice do women trust the most about pregnancy?
2. HEW? TBA? Health professionals? Relative? Why?
3. Which ones are the most useful: **Probe:** e.g. have the best skills and knowledge? Convenient? Close to home?
4. How do you support your wife during pregnancy and delivery?

**Perceptions of HEWs service quality**

1. How do you feel about the maternal health services provided by HEW in this community? **Probe:** for availability, adequacy, distance, usefulness, and limitations. How do you feel about the skills and knowledge of HEWs? What are they good at? How are their maternal health skills?
2. How do you feel about their attitude towards the community? Ask for examples.
3. What would you like to see improved?
4. What services do you expect to be delivered to mothers by the HEWs?
5. Who do you feel should be present when you women give birth? (**Probe:** family, TBA, HEW, midwife?) Why?

**Probe:** comfort, profession person with skills in childbirth?

1. Last year there was a community mobilization about maternal health and institutional delivery, do you remember it? Can you remember talking with the HDA leaders about this?

**Probe:** Can you remember what they told you? What do you think about it?

1. For those of you who are HDA leaders: how did your community respond? How can we improve the messages to families about maternal health?
2. Do HEWs and other providers use mobile telephones or other technology to coordinate with health centres and other MH services? Do you think this important? Why?
3. What do you think the Government can do to improve the maternal health services (**probe** at levels: health facilities, district health office and zone health department)?
4. Do you think there is enough infrastructure, materials and logistic to give an adequate and high quality MH service?

**REACH Ethiopia, REACHOUT Project Context Analysis Study Key Informant Interview Topic Guide with Kebele Administrator (KII_KA)**

***Instruction for Interviewer***

Take consent

Fill in the information sheet

Information sheet KII-KA

| **Respondent identifier** |  |
| --- | --- |
| **Gender** |  |
| **Educational level** |  |
| **What is your role in this Kebele** |  |
| **For how long did you serve in this Kebele** |  |

**MH services**

1. What maternal health services are available in your Kebele?

**Probe:** Where are these services? (Health centres, health posts). Who provides these services (TBA? HEW?). When and how often do pregnant women receive these services?

1. Do you think all the maternal health services that women need are available in your community? What other services are needed?
2. What is your role in facilitating quality maternal health service in the community?

**Maternal health and HEW program**

1. In your community where do women choose to get MH services?

Probe: Health Post? HC? Or from TBA or HDA. Why?

1. Why do you think some pregnant women in your community deliver in the health facilities? Why do other women decide to deliver at home? Do you think there is a difference in delivering at home and HC? What kind of difference?
2. Do you think the HEW program has changed the maternal health service coverage? (Utilization, quality,) in what way? Ask for examples.
3. Are there some women in your community who don’t have access (physical, financial) to maternal health advice (from HEW or other providers)? How are they getting the maternal health service?
4. Does your Keble have special support for women’s who can’t afford to get MH during referral? What kind of support?
5. Are there limitations to the maternal health services provided by HEWs? Are they available 24 hours? Does their MH work sometimes not go well? Why do you think that is?
6. Are there things that go well in maternal health services provided by HEW? Can you give an example? What makes this success?
7. What do you think are the main challenges in maternal health services?
8. What do you think are the main reasons for maternal mortality in this district? How do you think maternal mortality can be reduced?

**Monitoring and Evaluation**

1. How do you monitor and evaluate the HEWs program? (**Probe:** Plan, Report, using data for decision making)
2. Do you have meeting with HEWs regularly? How often? What do you discuss during that meeting?
3. In what way do you support HEWs program while they are giving MH services? (**Probe**: Do you have supervision program? How often? What do you do? Is there feedback mechanism)

**Perceptions of service quality**

1. What is the importance of the work done by HEWs?
2. How do you feel about the maternal health services provided by HEW in this community?

**Probe:** for availability, adequacy, and distance to HC, usefulness, and limitations.

1. How do you feel about the skills and knowledge of HEWs? What are they good at? How are their maternal health skills?
2. How do you feel about their attitude towards the community? Ask for examples.
3. What do you think is the community perception regarding the HEWs service on MH?
4. Whom do you think the community chooses to seek for an advice or to get service? Why?
5. What services do you expect to be delivered to mothers by the HEWs?
6. How does referral to the health centre happen (by whom?) are there any costs?
7. What do you think the role of the Keble Administrator should do to improve the maternal health services (**probe**: at levels: health facilities, district health office and zone health department)?
8. Do you think there are enough infrastructures, materials and logistic to give MH service is adequate to give high quality MH service? If no; why not?

**Health seeking behaviour**

1. In your community Normally what does a woman do when she finds out she is pregnant

**Probes**: Go to health post? Get a test? Where?

1. What do women do to make sure their pregnancy safe?

**Probes**: where can do they seek advice? From whom? (Relatives? TBA? HEWS? Health professionals? Other members of the community? Leader of HDA?) What sort of person is preferable?

1. Who or what are the most important influences on the services a woman accesses during pregnancy? **Probe:** TBA? Family? HEW? Media (radio etc)?
2. In this area the proportion of women receiving ANC is quite high, but the proportion of women choosing institutional delivery is low, why do you think this is?
3. From your experience where do you think women in your Keble commonly go to give birth? How do they decide where to give birth? How can they be encouraged to give birth in a health facility?

**Probes:** if at health centre, how can you get there? Who helps you? How long does it take?

1. Last year the HDA did community mobilization to promote institutional delivery, how did they do this in your Kebele?
2. What was the impact of the community mobilization?
3. Do you have ideas for additional activities that would help?

**Probe:** what about increased communication and monitoring of pregnant women between HEWs and other maternal health services, using mobile phone technologies?

**REACH Ethiopia, REACHOUT Project Context Analysis Study Key Informant Interview Topic Guide with Health Centre Head (KII-HCH)**

***Instruction for Interviewer***

Take consent

Fill in the information sheet

Information sheet KII-HCH

| **Respondent identifier** |  |
| --- | --- |
| **Gender** |  |
| **Educational level** |  |
| **What is your profession** |  |
| **For how long did you work in the Health centre?** |  |

**Perceptions of HEWs services**

1. What services are available for MH services in your health centre?

**Probe**: Who provides these services (midwife or other clinical nurses)? When and how often do pregnant women receive these services?)

1. How do you feel about the maternal health services provided by HEW in this community? **Probe:** for availability, quality, adequacy, distance, usefulness, and limitations.
2. How do you assist the HEW’s in providing quality MH services in the community?
3. How do you see your communication/relation with HEWs?

(**Probe:** Is it strong? Weak? In what way you want to improve it?)

1. Is there referral linkage between HEWs and HC?

**Probe:** how do you receive? Written? Oral? See if there is written referral slip

1. What challenges does HEW have in making referrals? How could the referral mechanism be improved?
2. What services do you expect to be delivered to mothers by the HEWs?
3. What is your role in facilitating quality maternal health service in the HC/community?
4. Where do you think women would like to give birth (TBAs, HPs/HCs)? Why?
5. Is there payment for MH services? (For which? How much? Did you take this as a challenge woman’s not to seek maternal health service?)

**Monitoring and Evaluation**

1. How do you monitor and evaluate the MH service delivered by HEWs? (**Probe:** Plan, Report, using data for decision making)
2. In what way do you support HEWs program while they are giving MH services? (**Probe**: logistic? Materials? Transportation for community work?)
3. Do you have supervision program? (**Probe:** How often? What do you do? Is there feedback mechanism?)
4. How do you monitor women’s satisfaction on MH services in the health facility? (**Probe:** do you have suggestion box, book or interview clients? What do they say? Do you check the suggestions and make change?)
5. What do you think is women’s perception regarding the service your health facility is giving?
6. Do you have regular meeting with HEWs? (How regular? Why do you meet? Probe: plan, report, data for decision making)
7. In this area the proportion of women receiving ANC is quite high, but the proportion of women choosing institutional delivery is low, why do you think this is?

**Probe**: Why do you think some pregnant women in your community deliver in the health facilities? Why do other women decide to deliver at home?

1. How do you feel about the idea of HEWs using mobile technology to link their services to the HC and to monitor their work?
2. Do you think the HEW program has changed the maternal health service coverage? (Utilization, quality,) in what way? Ask for examples.
3. What is the importance of the work done by HEWs?

**Perceptions of service quality**

1. How do you feel about the skills and knowledge of HEWs? What are they good at? How are their maternal health skills? Is there anything done at your facility to support them? Ask for example
2. How do you feel about their attitude towards the community? Ask for examples.
3. What would you like to see improved?
4. What do you think is your role as a health centre head you should do to improve the maternal health services (**probe:** at each level district health office, zone health department and RHB)?

**REACH Ethiopia, REACHOUT Project Context Analysis Study Key Informant Interview Topic Guide with Delivery Case Team Leader (KII-DCTL)**

***Instruction for Interviewer***

Take consent

Fill in the information sheet

**Information S**heet KII-DCTL

| **Respondent identifier** |  |
| --- | --- |
| **Gender** |  |
| **Educational level** |  |
| **What is your profession** |  |
| **For how long did you work in this Health centre** |  |

**Perceptions of HEWs services**

1. What services are available for MH services in your health centre?

**Probe**: When and how often do pregnant women receive these services?

1. What is your role in providing MH services?
2. How do you feel about the maternal health services provided by HEW in this community? Probe for availability, quality, adequacy, distance, usefulness, and limitations.
3. How do you assist the HEW’s in providing quality MH services in the community?
4. How do you see your communication/relation with HEWs?

(**Probe:** Is it strong? Weak? In what way you want to improve it?)

1. What services do you expect to be delivered to mothers by the HEWs?

**Perceptions about maternal health**

1. What is your role in facilitating quality maternal health service in the HC/community?
2. Where do you think women would like to give birth (TBAs, HPs/HCs)? Why?
3. Is there payment for MH services? (For which? How much? Did you take this as a challenge for women’s not to seek maternal health service?)
4. Why do you think are the reason women come for ANC follow up and they don’t come for delivery? (the number is not consistent) What needs to be done to improved?

**Monitoring and Evaluation**

1. In what way do you support HEWs program while they are giving MH services? (**Probe**: technical assistant, training, orientation?)
2. Do you have supervisory role to support the MH program at health post level? (**Probe:** How often? What do you do? Is there feedback mechanism?)
3. How do you monitor women’s satisfaction on MH services in the health facility? (**Probe:** do you have suggestion box, book or interview clients? What do they mostly say? Do you check the suggestions and make change?)
4. How do you think we can improve the performance of HEWs in maternal health services?
5. What do you think is women’s perception regarding the MH service your health facility is giving?
6. Do you have regular meeting with HEWs? (How regular? Why do you meet? Probe: plan, report, data for decision making)
7. In this area the proportion of women receiving ANC is quite high, but the proportion of women choosing institutional delivery is low, why do you think this is?
8. How do you feel about the idea of HEWs using mobile technology to link their services to the HC and to monitor their work?
9. Why do you think some pregnant women in your community deliver in the health facilities? Why do other women decide to deliver at home?
10. What is the importance of the work done by HEWs?

**Perceptions of service quality**

1. Have you participated in giving training for HEW’s on Clean and safe delivery

**Probe**: how did it go? Do you think the time allotted for the training is adequate? How do you see the performance of the HEWs in getting the skill? Are you confident to tell that HEWs can do their work independently? If not why?

1. Aside from the delivery, how do you feel about the skills and knowledge of HEWs on other MH services? What are they good at? Is there anything done at your facility to support them? Ask for example
2. How do you feel about their (HEWs) attitude towards the community? What do you hear from community about the service they get at HP level? Ask for examples.

**Referrals and management**

1. Have you received any referral in the last 2 months? Is it orally or written? How many? What kinds of cases are commonly referred from HEWs? Do you give feedback to the HEW?
2. Have you recently faced complicated cases with delay of referral by HEWs or TBAs? What happened?
3. How do high risk mothers come to the health facility (local transportation, ambulance? do they come escorted by HEW/TBAs or they come alone??)
4. Do you get other referrals other than HEWs? (**Probe:** TBAs or HDA)
5. What would you like to see improved?
6. What do you think is your role as delivery case coordinator you should do to improve the maternal health services?
7. How do you see your communication/linkage with HEWs? Do you think you have strong connection or weak? What would you like to improve?

**REACH Ethiopia, REACHOUT Project Context Analysis Study In-depth Interview Topic Guide with Traditional Birth Attendant (IDI-TBA)**

***Instruction for Interviewer***

Take consent

Fill in the information sheet

**Information sheet IDI-TBA**

| Respondent identifier |  |
| --- | --- |
| What is your age? |  |
| How many years education did you receive | None, Primary 1-3; primary 4-6;  Jun secondary ….yrs; senior secondary …yrs  Tertiary ………yrs |
| What is your role in this community? | (e.g. HDA) |
| Title of CTC provider | TBA |
| How many deliveries do you assist each year |  |
| Are you receiving an income from this work? | Yes/No  If, how much per delivery?  ………………………… local currency |
| Have you any training for delivery? | Describe |

**Introduction**

1. Please introduce yourself, and tell us how you became a TBA?
2. How long have you been working as a TBA? And why did you decide to do this type of work?
3. Do you have any other occupation? What is it?

**Maternal health work**

1. What services or advice do you currently provide for pregnant women and delivering mothers?
2. How do you approach a pregnant woman? What do you do first? How?

**Probe:** do you wait until they visit you themselves??

1. Are you confident that you have the skills to provide a high quality service at delivery?
2. Who do you have contact with during your work as a TBA?

**Probe:** HEWs? HC midwives? HDA? Others?

1. Under what circumstances do you contact the HEW?
2. How is your communication with the HEW?
3. Do you think HEWs are well equipped to do their work in maternal health?
4. Do HEWs have the right skills for delivery services? What other services do they do?
5. What goes well and what doesn’t go so well in the work of an HEW and why?

**Facilitators and Barriers for maternal health work**

1. What thing can be improved to give women higher quality maternal health services?
2. What do you think does not always go well in your work? Can you give an example?
3. What do you think about the quality of care in general for MH services provided in the community?

**Referral**

1. What do you do when a pregnant woman has a problem you cannot solve? Who do you refer to? How does the referral work?
2. What is the referral mechanism for high risk pregnant women?

**Probes:** Are there any difficulties in making referrals? (Transport, costs, referral system, distance, attitudes of clients?)

1. Do you refer women to HEW? When?

**Probe:** Only if they have a problem? If they are high risk? Always?

**Community attitudes to MH**

1. Who do women normally contact when they are pregnant? At what stage of pregnancy do they normally seek health advice?
2. Do you think that women should seek ANC? At what stage of pregnancy?

Probe: Are there any difficulties in encouraging women to seek ANC? What? How can they be encouraged to go at the right time?

1. Why do women choose to call you during labour?
2. What does the community think about the need for MH services?

**Probe**: home? Health post? Health centre? Why? Attended by whom? TBA, HEW?

1. Do you face challenges in encouraging high-risk women that they should deliver in a health facility?

**Probe:** Why? What/who influences the decisions women make about their maternal health seeking

1. Do you think it's important for women to get advice after delivery? When? From whom? what kind of advise should be given

**Probe:** about nutrition, hygiene, breast feeding, family planning.

1. What can be done to improve women’s maternal health seeking?

**Probe**: trust, education, community activities

1. How do you communicate with community members on maternal health issues

**REACH Ethiopia, REACHOUT Project Context Analysis Study Key Informant Interview Topic Guide with District HEP Coordinator (KII-HEP/D)**

***Instruction for Interviewer***

Take consent

Fill in information and recording sheet

Explain process

Information sheet KII-HEP/D

| Respondent identifier |  |
| --- | --- |
| Sex HEP coordinator |  |
| Year served as HEP coordinator |  |
| Educational level |  |

**Perceptions of provider services**

1. Please tell us about your work
2. What has been your involvement with HEP? How do you support HEP?

Supervision, training, guidance, provision of Supplies.

1. What services are available for mothers in your district?

**Probe**: Who provides these services (TBA? HEW? Health workers?). When and how often they get?)

1. How do you feel about the maternal health services provided by HEW in HPs?

**Probe** for availability, distance, and usefulness, and limitations, quality, skill

1. What would encourage mothers to go antenatal care with a HEW?
2. Are all the maternal services that women needed available at HPs? What other services are needed?
3. What services do you expect to be delivered to pregnant women by the HEWs at HP level?

**Perceptions about maternal health**

1. How do you see the importance of providing maternal health service at HP level? (Decrease maternal mortality, community utilization service, increase referral links.)
2. What MH services are available for women in the health centres? And the health posts?
3. What makes some women decide to attend ANC with HEWs, some in health centres and others to seek advice from TBAs?

**Probe** (service quality, cultural belief, skill and knowledge of providers, family advice, costs, time)

1. Why do you think some pregnant women deliver in the health facilities? Why do other women decide to deliver at home?
2. Do you think the number of mothers attending ANC matches with those attending institutional delivery? If not; why?
3. Are there some women who don’t have access to maternal health advice in your district? Why?
4. How do you see the role of HEWs in providing maternal health services?

**Probe:** Are there limitations to the maternal health services provided by HEWs? Does their MH work sometimes not go well? Why do you think that is?

1. What maternal health work HEWs do well? Why?
2. What maternal health work HEWs do not do well? Why? How can it be improved?
3. What do you feel about the different maternal health service providers?

**Probes:** TBA, HEWs, health centre midwives. How useful are the different providers’ services?

**Monitoring and evaluation**

1. How would you support HEP? (supervision, training, reporting, feedback ...)
2. How the integration between health centre and health post strengthened? (training by HC to HEWs, supply of logistics, reporting, meetings)
3. How would the HEWs get logistic and supplies for MH services (frequency, stock outs, types)
4. Do you have a meeting with HEWs (how frequent, what agendas discussed...)
5. In this area the proportion of women receiving ANC is quite high, but the proportion of women choosing institutional delivery is low, why do you think this is?
6. How do you feel about the idea of HEWs using mobile technology to link their services to the HC and to monitor their work?

**Perceptions of service quality**

1. How do you feel about the skills and knowledge of HEWs? What are they good at? How are their maternal health skills?
2. What would you like to see improved?
3. How do you see the quality of MH services provided by HEWs?
4. How do you see the availability of guidelines and manuals at HP to support HEWs (type, number, language they are written...?)
5. How do you evaluate the trainings provided to HEWs to provide MHs (duration, type, scope) and how can be improved?

**Motivation**

1. How do you see the motivation of HEWs to provide MH services?
2. What do you think motivates and de motivates HEWs to provide MH services? (salary, continuing education, transport and other infrastructures, logistics
3. What do think the government should do to motivate HEWs?

**Referral**

1. How is the referral system being coordinated in you districts? (Community to HP, from HP to HC and HC to hospital)
2. What support is given by the Woreda to the referred mothers for maternal health service? How it is organized?
3. What problems affecting the referral system in your district? (Infrastructure, cost, fuel, budget, transport.)
4. Do HEWs and other providers use mobile telephones or other technology to coordinate with health centres and other MH services? Do you think this important? Why?
5. What do you think the Government can do to improve the referral system?

**REACH Ethiopia, REACHOUT Project Context Analysis Study Key Informant Interview Topic Guide with Zonal/Regional HEP Coordinator (KII-HEP/Z/R)**

***Instruction for Interviewer***

Take consent

Fill in information and recording sheet

Explain process

**Information sheet**

| **Gender** |  |
| --- | --- |
| **Educational level** |  |
| **What is your profession** |  |
| **For how long did you work in this position** |  |

**Background**

1. Can you tell me a bit about your work in relation to MH services?
2. What has been your involvement with HEP?
3. Are you in direct contact with HEWs? If in direct contact what is your role?
4. What do you think goes really well in HEP? Give examples? What things help to make this go well?
5. What do you think does not always go well? Can you give an example? What things are influencing that this does not go well? Can you give an example?
6. Are you familiar with the planning of HEP? If yes, how is this organised? Probe for population provider ratio; criteria/process for the selection of areas where they are used? Process for task identification and legislation of tasks, workload assessment, integration with health system.
7. What do you think about each of these measures? If you HDA to decide what should happen for a new programme what would you include and what would you do different?
8. How are HEWs recruited and the criteria for selection? What are their incentives, remuneration, career perspectives, training, continuing education, supervision?
9. What things influence job satisfaction and motivation of HEWs and how? What motivates or de motivates them?

**Perception of MH service**

1. What do you think is the importance of HEP in implementation of MH services? What is their potential contribution towards MH services? Do you see any downsides of this MH programme?
2. What can be done to improve the maternal health service? What would you suggest? How could this be done?
3. What policies, strategies, guidelines for HEP are you aware of related to MH services? What are the most important aspects of these policies in your opinion? What are strong points in the MH policies what could be improved? (Clean and safe delivery training, and other maternal health related trainings?)
4. What do you think about the readiness of Health post in providing MH services ( infrastructure, materials, equipment)

**Referral**

1. How referral of is MH services are organized? (What do you think about the integration of the HP to HC, anything to be done to improve?)
2. What goes well and not so well in referral? Why? Examples?
3. How is the communication and interactions with colleagues (all cadres including supervisors, in charges, volunteers and TBAs organised?
4. How is this information about performance of programmes collected? What communication channels are used? What happens with this information? Do you give feedback about the results of the work? If so, how is this communicated by whom?
